# Risk prediction of atrial fibrillation progression in patients with paroxysmal atrial fibrillation: data from the RACE V study

**DOI:** 10.1016/j.ijcha.2026.101932

**Published:** 2026-04-20

**Authors:** D.K. Baron, M Samuel, H.J.G.M. Crijns, R.G. Tieleman, M.E.W. Hemels, U Schotten, D. Linz, I.C. Van Gelder, M. Rienstra, for the RACE V Investigators

**Affiliations:** a Department of Cardiology, University Medical Centre Groningen, Groningen, The Netherlands; b Department of Medicine, Dalhousie University, Halifax, Canada; c Department of Cardiology, Maastricht University Medical Centre+, Maastricht, The Netherlands; d Cardiovascular Research Institute Maastricht (CARIM), Maastricht University, Maastricht, The Netherlands; e Department of Cardiology, Martini Hospital, Groningen, The Netherlands; f Department of Cardiology, Rijnstate Hospital, Arnhem, The Netherlands; g Department of Cardiology, Radboud University Medical Centre, Nijmegen, The Netherlands; h Department of Physiology, Maastricht University, Maastricht, The Netherlands

**Keywords:** Atrial fibrillation, Paroxysmal AF, AF progression, Risk prediction, Continuous rhythm monitoring, Implantable loop recorder

## Abstract

**Background:**

Atrial Fibrillation (AF) may progress from paroxysmal AF (PAF) to more sustained forms, but predicting which patients progress remains a challenge. The RACE V study is a prospective, observational study aiming to characterize phenotypical differences between patients with and without AF progression. Based on interim data of the RACE V study, a clinical risk prediction model for AF progression in patients with PAF was previously developed. The aim of the current analysis is to assess its performance in the complete cohort over extended follow-up.

**Methods:**

In the RACE V study, 612 patients with PAF were extensively phenotyped and continuously monitored using implantable loop recorders to track AF recurrences. AF progression was the primary outcome, defined as (1) progression to persistent or permanent AF, or (2) AF burden increase > 3%, during complete follow-up. The risk score incorporates five clinical predictors at baseline: sex, PR interval duration, left atrial contractile function, waist circumference, and presence of mitral valve regurgitation. Prediction model performance was assessed using receiver operating characteristic (ROC) curve-derived area under the curve (AUC).

**Results:**

Patients had a median age 64 [57 – 70] years, and 42% were female. During a follow-up of 3.4 (2.8 – 3.7) years, 108 (5.2%/year) patients progressed. The risk prediction model demonstrated a C-statistic of 0.647 (95% CI: 0.590–0.707**)** in the full cohort, compared with 0.709 (95% CI: 0.617–0.801) in the interim cohort (DeLong’s unpaired test, p = 0.656).

**Conclusions:**

The model may help clinicians identify patients at risk of progression, showing stable performance in the whole cohort over extended follow-up.

## Highlights

• Predicting AF progression in Patients with Paroxysmal AF (PAF) remains a challenge.
• A simple risk model was previously developed in RACE V based on interim data.
• This study validates the model in the full cohort with extended follow-up.
• The risk score showed moderate discriminatory power (C-statistic 0.65).
• This practical tool that may help clinicians identify patients at risk of progression.

## 1 Introduction

Atrial fibrillation (AF) may progress from paroxysmal AF (PAF) to more persistent forms, and progression is associated with increased disease burden [1], [2], [3], [4]. Predicting which patients will progress remains challenging, but may help guide management decisions and prevent adverse outcomes. In an interim cohort of the Reappraisal of AF: Interaction Between HyperCoagulability, Electrical Remodelling, and Vascular Destabilisation in the Progression of AF (RACE V) study, an AF progression risk prediction model was developed and published [5]. This model was based on 417 patients with PAF, who were extensively phenotyped and underwent continuous rhythm monitoring. The risk prediction model incorporates five clinical predictors at baseline (sex, PR interval duration, left atrial contractile function, waist circumference and presence of mitral valve regurgitation; Table 1) to estimate an individual’s risk of AF progression at two years, having achieved a C-statistic of 0.709 (95% CI: 0.617 – 0.801). The current study aims to assess the performance and validate the model in the complete RACE V cohort, which includes 204 patients outside the derivation cohort, and over an extended follow-up period of 3.4 years (end of study).

**Table 1:** The RACE V clinical risk score.

**Sex**	**Points**
Female	−2
Male	0
**PR interval** (ms)	**Points**
≤122	0
123–148	1
149–174	2
175–200	3
201–226	4
227–252	5
253–278	6
279–304	7
305–330	8
331–356	9
>356	10
**Left atrial contractile function** (%)	**Points**
≤12	3
13–17	2
18–22	1
23–27	0
28–32	−1
>32	−3
**Waist circumference** (cm)	**Points**
≤84	0
85–96	1
97–108	2
109–120	3
121–132	4
>132	5
**Mitral valve regurgitation**	**Points**
Yes	5
No	0

**Risk estimation of AF progression at 2.0**–––**3.5 years based on total points**											
**Total points**	0	1	2	3	4	5	6	7	8	9	10
**Risk %**	2.3	3.1	4.2	5.6	7.5	9.9	13.0	16.9	21.6	27.2	33.7

## 2 Methods

### 2.1 Study design and population

The RACE V study has been previously described [6]. The RACE V study was a Dutch, multicenter, investigator-initiated, prospective observational study, which aimed to characterize phenotypical differences between patients with and without AF progression. Eligible patients had > 2 documented episodes of self-terminating AF, or 1 documented episode of AF and 2 + symptomatic episodes suspected to be AF. Patients with history or planned pulmonary vein isolation (PVI), currently on amiodarone treatment, and/or not willing to undergo implantable loop recorder (ILR) implantation were excluded. All included patients were continuously rhythm monitored using ILRs or pacemakers with an atrial lead and equivalent AF detection algorithms, allowing for precise tracking of AF recurrences. The primary outcome of the study was AF progression, defined as (1) progression to persistent or permanent AF, or (2) AF burden increase > 3%, over complete follow-up. AF progression was adjudicated by three experts (I.C.V.G.; M.R.; M.S.). All patients provided written informed consent. The RACE V study was conducted in accordance with the Declaration of Helsinki, and the study protocol was approved by the Medical Ethics Review Committees of the University Medical Center Groningen and all participating centers.

### 2.2 Clinical assessment

At baseline, clinical history, symptoms, and current medications were recorded. A physical examination, 12-lead electrocardiogram (ECG), echocardiography, vascular assessment, and cardiac computed tomography (CT) were performed, and blood samples were collected.

### 2.3 Statistical analysis

Continuous data are presented as median (interquartile range), and categorical data as counts (%). The risk model was originally developed using stepwise multivariable logistic regression analyses for the primary outcome using baseline variables, and was bootstrapped with optimism correction [5]. Performance of the RACE V clinical risk score in the complete cohort was assessed by plotting a receiver operating characteristic (ROC) curve for the primary outcome and calculating the area under the curve (AUC), with 95% confidence intervals estimated using nonparametric bootstrapping. Model calibration was evaluated using calibration plots. Between-group AUCs were compared using DeLong’s unpaired test. Imputation was implemented for missing values using the R package MICE. Analyses were performed using R statistical software (v4.4.1; R Core Team, 2021). A two-sided p-value ≤ 0.05 was considered statistically significant.

## 3 Results

A total of 612 patients with PAF were enrolled. Patients had a median age of 64 (57 – 70) years, and 42% of patients were female. At baseline, 180 (29%) patients had heart failure, 521 (85%) had hypertension, 61 (10%) had coronary artery disease, and 50 (8%) had diabetes. The median CHA_2_DS_2_-VA score was 2 (1 – 3). During a median follow-up of 3.4 (2.8 – 3.7) years of continuous rhythm monitoring, 108 (5.2%/year) patients showed AF progression. Sixty-seven (11%) patients had no recurrent AF after inclusion.

**Fig. 1:**
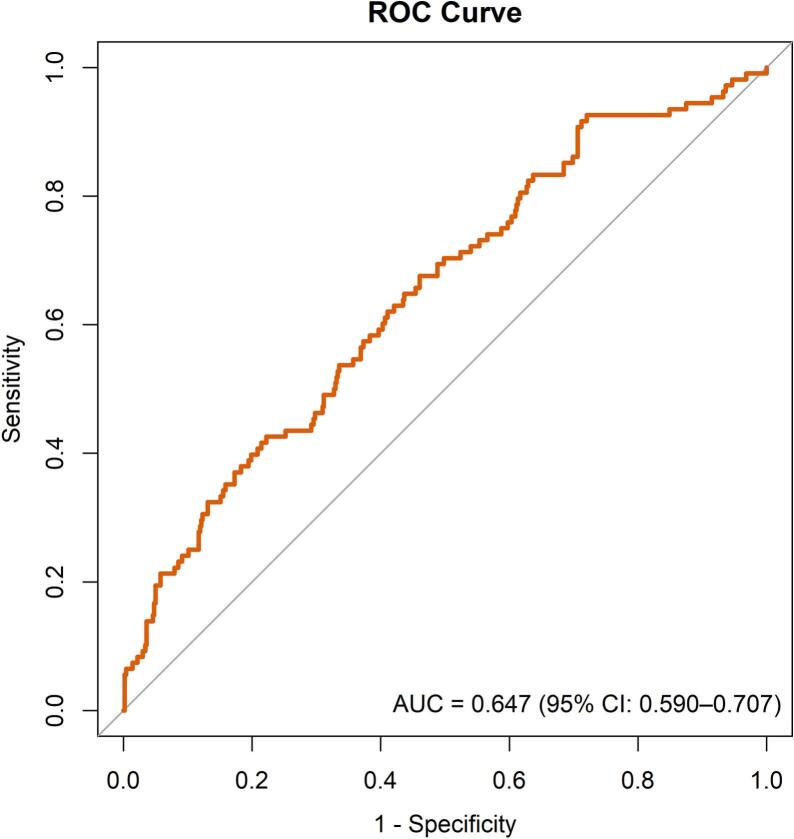
ROC curve for prediction of AF progression in RACE V. AF, atrial fibrillation; AUC, area under the curve; ROC, receiver operating characteristic.

The AF progression risk prediction model demonstrated a C-statistic of 0.647 (95% CI: 0.590 – 0.707; Fig. 1) for the primary outcome in the entire cohort at complete follow-up (DeLong’s unpaired test p value = 0.656 relative to the interim cohort). The calibration plot and associated performance metrics are presented in Suppl. Fig. S1 and Suppl. Tables S1 and S2.

The cohort comprised of 408 patients that overlapped among the interim and full cohort, and 204 patients not included in the derivation cohort (*see* Suppl. Fig. S2). In a longitudinal analysis of the 408 overlapping patients, the model yielded a C-statistic of 0.711 (95% CI: 0.610 – 0.799) at interim follow-up, and 0.675 (95% CI: 0.595 – 0.748) at full follow-up (DeLong’s unpaired test p value = 0.560; Suppl. Fig. S3).

Sensitivity analyses were performed in the 204 patients not included in the derivation cohort, yielding a C-statistic of 0.585 (95% CI: 0.474 – 0.692) over full follow-up (DeLong’s unpaired test p = 0.329 relative to the complete cohort; Suppl. Fig. S4*;* Suppl. Table S3).

## 4 Discussion

The RACE V clinical risk score demonstrates moderate discriminatory power in the complete cohort of 612 patients with PAF over 3.4 (2.8 – 3.7) years of follow-up. The performance of the model remained consistent with the interim analysis while using a larger cohort over an extended follow-up, including in sensitivity analyses restricted to patients outside the derivation cohort; representing partial internal validation and providing additional evidence of the model’s stability within this population.

The model was designed to be simple and practical, relying on routinely collected clinical variables that are available in most standard care. A decrease in the C-statistic was observed at 3.4 years (0.65 vs. 0.70 at 2 years), that may reflect limitations in the C-statistic which does not inherently account for varying follow-up times. Extending the prediction window beyond the original two years used to develop the score may have altered the event distribution, increased competing risks (like death and comorbidity presence), and/or weakened the association between baseline predictors and the primary outcome. However, comparative ROC curve analyses showed no appreciable difference in discrimination over the extended follow-up, indicating temporal stability during the period assessed.

Calibration analyses indicated a systematic tendency towards overestimation of absolute risk, particularly at higher predicted probabilities, with comparatively better calibration in the low-to-intermediate risk range, where the majority of patients were classified. The observed miscalibration suggests that the score may be better suited for relative risk stratification rather than for precise estimation of individual absolute risk.

In the broader context of clinical prediction tools for AF progression, similar scores have generally shown modest discrimination. For example, the HATCH score was developed to predict progression from paroxysmal to sustained AF, and demonstrated modest predictive value with C statistics in the range of 0.62–0.68 in external cohorts, only marginally outperforming simple age or comorbidity measures [7], [8]. Common clinical risk scores such as CHA_2_DS_2_–VASc have likewise shown modest discrimination for rhythm outcomes [9]. While direct comparisons in our cohort were beyond the scope of this analysis, these findings indicate that moderate discriminatory performance is a common feature of clinical AF progression models and underscore the challenge of improving prediction with simple clinical variables alone.

The RACE V clinical risk score may help clinicians identify patients with PAF who are at increased risk of AF progression. Early identification could enable more timely intervention (like closer rhythm monitoring, targeted risk factor modification and earlier consideration of rhythm control strategies) to diminish AF progression or even reverse AF burden [[9], [10], [11], [12]]. However, the model requires external validation in an independent cohort before clinical implementation can be considered. To our knowledge, such a cohort with continuous rhythm monitoring using ILRs and thorough phenotyping is not yet available. The identification or development of such a cohort would be an important next step in assessing the model’s broader applicability and utility.

### 4.1 Strengths and limitations

A strength of our study is the comprehensive phenotyping at baseline and continuous rhythm monitoring, which provides a reliable basis for detecting AF recurrence; reducing the risk of misclassification of AF progression status.

There are several limitations to consider. First, external generalizability of the score remains limited, and formal validation in an independent cohort is required to confirm broader applicability. Second, treatment in the study was at the discretion of the treating physician, which may have influenced AF progression. Third, patient overlap between the derivation and evaluation cohorts means discrimination estimates may have been optimistic; however, bootstrap validation and analyses in non-derivation patients showed broadly consistent performance. Fourth, missing data were addressed through imputation, but consistent results were observed in complete-case-only analyses. Fifth, not all factors contributing to AF progression may have been systematically assessed in the study, but may contribute to progression. Lastly, the possibility of residual confounding cannot be excluded in an observation study like this one.

## 5 Conclusions

The RACE V clinical risk score offers a practice tool that may help identify patients at increased risk of AF progression. The risk score had stable performance over extended follow-up, supporting its use in identifying patients for more intensive monitoring or proactive management aimed at delaying and/or preventing AF progression.

**Trial Registration Number:** Clinicaltrials.gov Identifier NCT02726698.

## Data availability

The data that support the findings of this study are available from the corresponding author upon reasonable request.

## CRediT authorship contribution statement

**D.K. Baron:** Writing – original draft, Formal analysis. **M Samuel:** Writing – review & editing, Supervision, Methodology, Formal analysis. **H.J.G.M. Crijns:** Writing – review & editing. **R.G. Tieleman:** Writing – review & editing. **M.E.W. Hemels:** Writing – review & editing. **U Schotten:** Writing – review & editing. **D. Linz:** Writing – review & editing. **I.C. Van Gelder:** Writing – review & editing, Supervision, Methodology, Conceptualization. **M. Rienstra:** Writing – review & editing, Supervision, Methodology, Conceptualization.

## Funding

We acknowledge the support from the Netherlands Cardiovascular Research Initiative: an initiative with support of the Dutch Heart Foundation, CVON 2014–9: Reappraisal of Atrial Fibrillation: Interaction between HyperCoagulability, Electrical Remodeling, and Vascular Destabilisation in the Progression of AF (RACE V). Unrestricted grant support from Medtronic Trading.

## Declaration of competing interest

The authors declare the following financial interests/personal relationships which may be considered as potential competing interests: [**MS** reports receiving honoraria from the American College of Cardiology Foundation. **HJGMC** reports fees and honoraria for lectures, education, and scientific advice from Atricure, Medtronic and Armgo. **MR** reports consultancy fees from Bayer (OCEANIC-AF national PI), InCarda Therapeutics (RESTORE-SR national PI), and Novartis, paid to the institution; and speaker fees from Daiichi-Sankyo and Pfizer, paid to the institution. MR further reports unrestricted research grants from the Dutch Heart Foundation, conducted in collaboration with and supported by the Dutch CardioVascular Alliance (01–002-2022–0118 EmbRACE); from ZonMW and the Dutch Heart Foundation (DECISION project 848090001); from the Netherlands Cardiovascular Research Initiative with support of the Dutch Heart Foundation (RACE V, CVON 2014–9; RED-CVD, CVON2017-11); from Top Sector Life Sciences & Health to the Dutch Heart Foundation (PPP Allowance, CVON-AI, 2018B017); and from the European Union’s Horizon 2020 research and innovation programme (EHRA-PATHS, grant agreement 945260). All other authors declare no conflicts of interest].

## Appendix A Supplementary data

Supplementary Data 1 Supplementary analyses including calibration plots and tables, ROC curve analyses with AUC comparisons, and a cohort flow diagram illustrating patient inclusion.
