# Supplementary material for: Risk prediction of atrial fibrillation progression in patients with paroxysmal atrial fibrillation: data from the RACE V study
Source: Int J Cardiol Heart Vasc. 2026 Apr 20;64:101932. doi: 10.1016/j.ijcha.2026.101932 (PMC13122194; doi:10.1016/j.ijcha.2026.101932)
Supplement: Supplementary Data 1 — Supplementary analyses including calibration plots and tables, ROC curve analyses with AUC comparisons, and a cohort flow diagram illustrating patient inclusion. [file mmc1.docx]

**SUPPLEMENT**

**
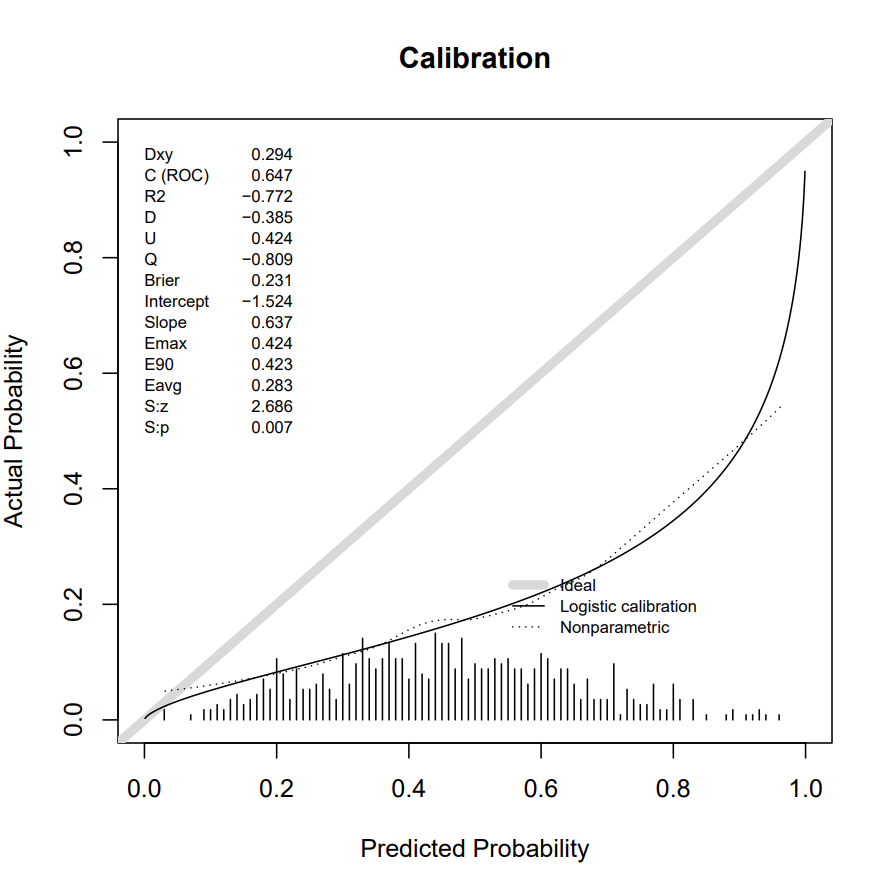
**

**Suppl. Figure 1** Calibration of predicted AF progression risk using the RACE V clinical risk score in the full cohort (n = 612).

**Suppl. Table 1** Summary calibration metrics for the full cohort (n = 612).

| Calibration-in-the-large (CITL) | 0.285 |
| --- | --- |
| Calibration slope | 0.637 |
| Brier score | 0.231 |

**Suppl. Table 2** Decile-level calibration for the full cohort (n = 612).

| **Decile** | **Mean predicted risk** | **Mean observed risk** | **Number of observations** |
| --- | --- | --- | --- |
| 1 | 0.164 | 0.097 | 62 |
| 2 | 0.257 | 0.032 | 62 |
| 3 | 0.330 | 0.164 | 61 |
| 4 | 0.381 | 0.164 | 61 |
| 5 | 0.434 | 0.115 | 61 |
| 6 | 0.479 | 0.230 | 61 |
| 7 | 0.535 | 0.197 | 61 |
| 8 | 0.593 | 0.148 | 61 |
| 9 | 0.661 | 0.246 | 61 |
| 10 | 0.792 | 0.377 | 61 |


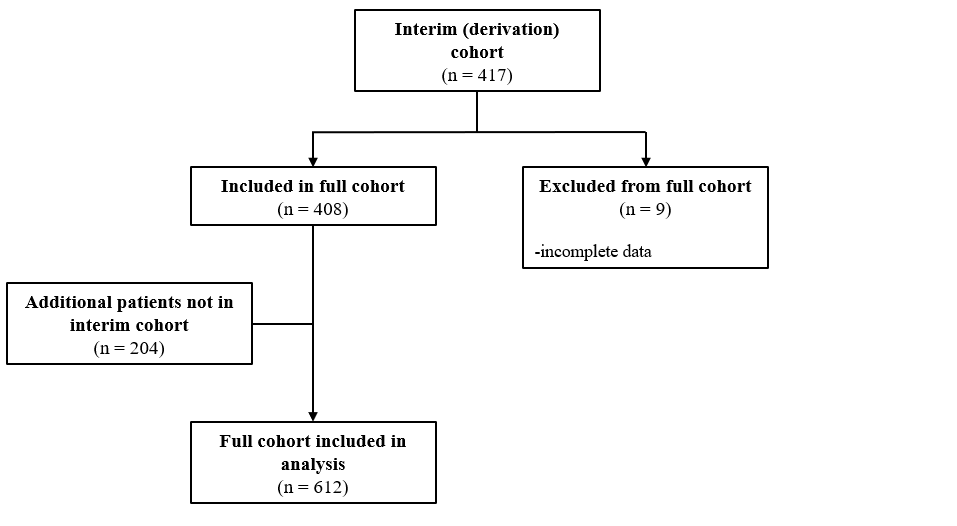


**Suppl. Figure 2** Flow diagram of cohort inclusion and analysis population.


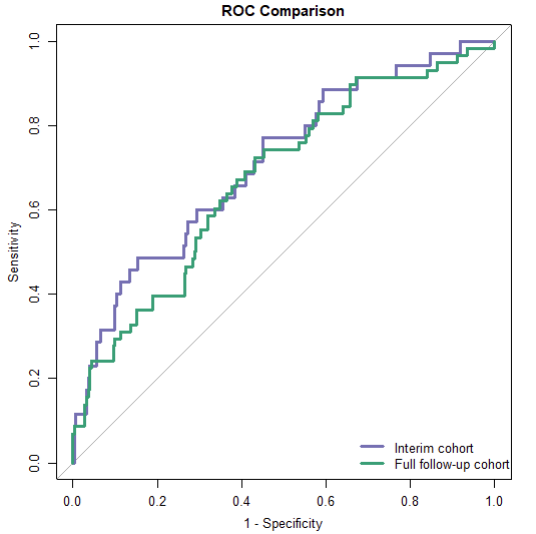


**Interim AUC** **=** 0.711 (95% bootstrap CI: 0.610 – 0.799)
**Full follow-up AUC** **=** 0.675 (95% bootstrap CI: 0.595 – 0.748)
**DeLong’s unpaired test p-value =** 0.560

**Suppl. Figure 3** Temporal comparison of ROC curves for AF progression prediction using the RACE V clinical risk score at 2-year interim and 3.5-year full follow-up in the same patients (n = 408) followed longitudinally. The score’s discrimination is stable over time. DeLong test (unpaired) was not significant.


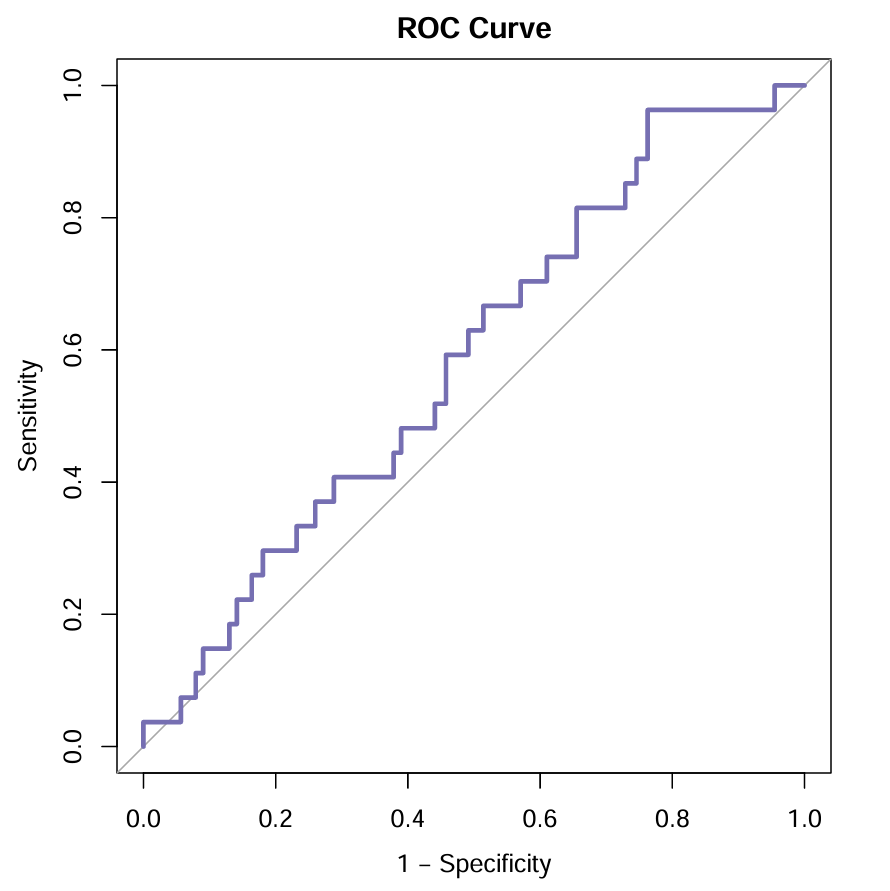

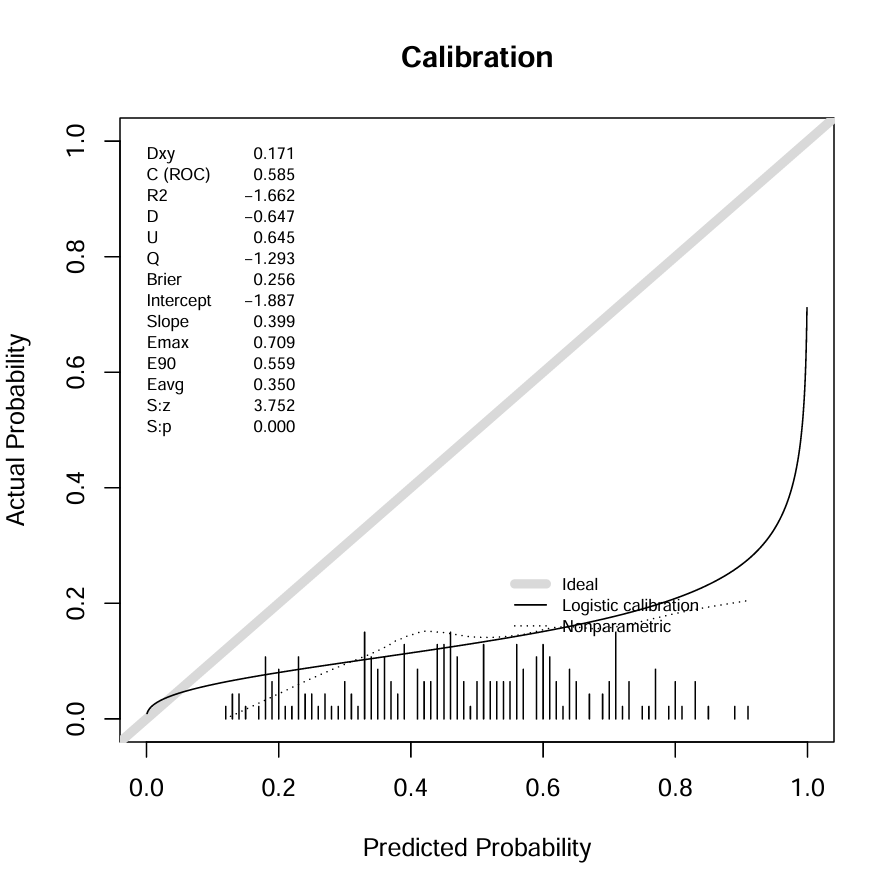


**` Non-overlap cohort AUC** = 0.585 (95% bootstrap CI: 0. 0.474 – 0.692)
**DeLong’s unpaired test p-value =** 0.329

**Suppl. Figure 4** ROC and calibration curves for AF progression prediction using the RACE V clinical risk score in non-overlap patients (n = 204). DeLong test (unpaired) relative to the full cohort (n = 612) was not significant.

**Suppl. Table 3** Summary calibration metrics for non-overlap patients (n = 204).

| Calibration-in-the-large (CITL) | 0.348 |
| --- | --- |
| Calibration slope | 0.399 |
| Brier score | 0.256 |
